# Dietary Exogenous α-Amylase Modulates the Nutrient Digestibility, Digestive Enzyme Activity, Growth-Related Gene Expression, and Diet Degradation Rate of Olive Flounder (*Paralichthys olivaceus*)

**DOI:** 10.4014/jmb.2303.03033

**Published:** 2023-06-29

**Authors:** Md. Tawheed Hasan, Hyeon Jong Kim, Sang-Woo Hur, Seong-Mok Jeong, Kang-Woong Kim, Seunghan Lee

**Affiliations:** 1 Core-Facility Center for Tissue Regeneration, Dong-Eui University, Busan 47340, Republic of Korea; 2 Department of Aquaculture, Sylhet Agricultural University, Sylhet-3100, Bangladesh; 3 Aquafeed Research Center, National Institute of Fisheries Science, Pohang 37517, Republic of Korea

**Keywords:** Aqua-feed, feed additive, olive flounder, digestive enzymes, alpha-amylase, digestibility

## Abstract

In this study, a 12-week feeding experiment was conducted to characterize the effects of exogenous α-amylase on the growth, feed utilization, digestibility, plasma α-amylase activity, feed degradation rate, and fecal particle size of olive flounder (*Paralichthys olivaceus*). Diet was supplemented with 0 (AA_0_; control), 100 (AA_100_), 200 (AA_200_), or 400 (AA_400_) mg/kg of α-amylase, respectively. Fish (273.1 ± 2.3 g) were stocked into 12 tanks (25 fish/1,000-L tank) and 3 tanks were randomly selected for each diet group. As a result, α-amylase was found to have no significant effects (*p* ≥ 0.05) on the growth, feed utilization parameters, and whole-body proximate compositions. α-Amylase-treated fish exhibited only a significant increase in the apparent digestibility coefficient of carbohydrates compared to the controls. In addition, in vitro analyses revealed that α-amylase dose-dependently increased (*p* < 0.05) the feed degradation rate, while photographs of the intestinal content after 2, 4, and 8 h of feeding demonstrated an improved degradation rate in the α-amylase-treated groups. Plasma α-amylase content was higher in the AA_200_ and AA_400_ groups, whereas the control group produced significantly larger-sized fecal particles (90% size class) than these two groups. In the intestine, no changes were observed in the expression levels of the immune-related TNF-α, IL-1β, IL-2, immunoglobulin-M, HSP-70, lysozyme, and amylase alpha-2A. However, growth-related genes IGF-1, IGF-2, TGF-β3, and growth hormone genes were upregulated in muscle tissues. Collectively, exogenous α-amylase has positive roles in the modulation of the digestibility coefficient, blood α-amylase concentration, growth-related gene expression, and diet degradation for improved digestion in olive flounder.

## Introduction

Olive flounder (*Paralichthys olivaceus*) is native to and distributed from the Western Pacific to the Kuril Islands and South China Sea [1]. In 2018, China, Korea, and Japan produced 57,567, 37,258, and 2,200 metric tons of olive flounder, respectively [2]. While carbohydrates are the primary sources of dietary energy for humans and domestic animals, Wilson, however, reported that although there is no specific requirement for carbohydrates in fish diets, incorporating carbohydrates into diet formulations reduces the protein and lipid catabolism, thereby saving energy and decreasing the synthesis of biological compounds in fish [3-5]. The carbohydrate enzymes, α-amylase, β-glucanase, and β-xylanase, increase the release of glucose, galactose, and xylose, respectively, from plant-based protein sources and enhance the energy digestibility in fish [6]. The results of carbohydrate incorporation studies are largely controversial. One study has reported that the inoculation of more than 12.6%dietary carbohydrate reduces the growth performance of rainbow trout (*Oncorhynchus mykiss*) [7], whereas many other studies have demonstrated that a higher percentage of carbohydrates enhances the protein and energy retention in Siberian sturgeon (*Acipenser baerii*) [8] and *O. mykiss* [9]. Therefore, exogenous digestive enzyme supplementation in fish diets has garnered increasing attention in the aquaculture of Yangtze sturgeon (*A. dabryanus*) [10] and cachama (*Piaractus brachypomus*) [11] as it promotes carbohydrate digestion and assimilation in these fish.

Similar to terrestrial animals, such as pigs and poultry [12], dietary exogenous enzymes can be used to reduce the impact of anti-nutritional factors in carnivorous fish diets containing plant-based feedstuffs [13]. Dietary supplementation with these enzymes increases the levels of substrates available to the intestinal microbial community, thereby improving nutrient digestion, synthesis of bioactive molecules, intestinal integrity, and fish growth [14]. Dietary administration of exogenous enzymes has been extensively studied in poultry and swine and applied in aquaculture feed to reduce both phytic acid levels and the anti-nutritional effects of non-starch polysaccharides to enhance the utilization of phosphorus and carbohydrates, respectively [15].

Several alternative animal- and plant-based sources have been proposed to satisfy the lipid and protein requirements of specific aquaculture species. However, the nutritional compositions of these alternative sources are often quite different from those of fish meal (FM) and fish oil (FO) [16]. Enhancing feed digestion and nutrient assimilation not only improves the well-being of fish but also increases their aquaculture profitability. The global carbohydrase market is dominated by xylanase, glucanase, and other commercially available carbohydrases, such as α-amylase, β-mannanase, α-galactosidase, and pectinase, which can hydrolyze carbohydrate polymers to produce low-molecular-weight oligosaccharides or polysaccharides [15, 17].

Amylase, an important endogenous digestive enzyme that degrades starch [18], is present in various fish species. Therefore, addition of exogenous amylase enzyme to diet formulations may facilitate the breakdown of complex carbohydrate polymers to produce glucose as an energy source in these fish [19]. Stone was the first to report that exogenous α-amylase supplementation in aquaculture feed increases the starch digestibility of silver perch (*Bidyanus bidyanus*) [4]. Later studies confirmed that dietary exogenous α-amylase significantly increases the dry matter digestibility and intestinal amylase activity in rohu carp (*Labeo rohita*) [19]. Dietary pepsin, papain, and α-amylase increase the weight gain (WG), feed conversion ratio (FCR), and protein efficiency ratio (PER) in Nile tilapia (*Oreochromis niloticus*) [20]. Natuzyme and Hemicell enhance the growth and blood parameters in Caspian salmon (*Salmo trutta*) [21]. Multi-enzyme complexes mainly consisting of fungal xylanase, cellulase, and glucanase improve the growth and feed utilization of African catfish (*Clarias gariepinus*) [22]. Administration of an enzyme cocktail comprising protease, β-glucanase, and xylanase significantly increases the apparent digestibility of *O. mykiss* [13]. Cellulase supplementation modulates the intestinal microbiome of grass carp (*Ctenopharyngodon idella*) [23]. However, exogenous enzyme administration has no significant effects on the growth and digestibility of *Oreochromis niloticus* [24] and *O. mykiss* [25]. To the best of our knowledge, the effects of dietary exogenous digestive enzymes, including α-amylase, on olive flounder have not yet been elucidated.

In this study, we aimed to identify and quantify the effects of different doses of exogenous α-amylase-inoculated diet (soybean meal: 10.3%, tapioca starch: 10%, and wheat flour: 11.5%) on the growth, feed utilization, and apparent digestibility coefficient (ADC) of *P. olivaceus*. We also estimated the alterations in their whole-body proximate composition, feed degradation, growth, immune-related gene expression, and blood amylase activity. Additionally, the fecal particle size and digestion in the stomach were also investigated after specific intervals in olive flounder.

## Materials and Methods

All experiments were approved by and conducted at the Aquafeed Research Center (Pohang), National Institute of Fisheries Science (NIFS), Republic of Korea, following the NIFS regulations on the Care and Use of Laboratory Animals (approval no. 2021-NIFSIACUC-07).

### Experimental Diet Formulation

Compositions of the experimental diets and proximate analyses results are shown in Table 1. Basal diets were prepared by thoroughly mixing the dry ingredients in an electric mixer, followed by extrusion in a twin-screw extruder (ATX-II; Fesco Precision Co., Korea) under the following conditions: feeder supply speed, 70 kg/h; conditioner temperature, 80°C; barrel temperature, 120–130°C; main screw speed, 650 rpm. The key ingredients for proteins (FM and soybean meal), carbohydrates (tapioca starch and wheat flour), and lipids (FO) in the experimental diets were purchased from Suhyup Feed Co. (Uiryeong, Korea). The required amount of α-amylase from *Aspergillus oryzae* (30 units/mg; Sigma Aldrich, USA) was dissolved in 75 ml of distilled water and sprayed over 1 kg of the extruded basal diet. Four diets were prepared with four different levels of α-amylase (AA): 0 (AA_0_), 100 (AA_100_), 200 (AA_200_), and 400 (AA_400_) mg AA/kg diet. Pellets were air-dried at 60°C for 3 h and stored at −20°C until use. All experimental diets contained chromium oxide (Cr_2_O_3_) as an inert digestibility marker.

### Fish Rearing and Feeding Trial

Juvenile *P. olivaceus* were collected from a private hatchery (Geoje-si, Gyeongsangnam-do, Korea). Prior to the start of the feeding trial, all fish were fed the AA_0_ diet for two weeks to acclimatize them to the experimental conditions and facilities. Then, olive flounders (initial weight 273.1 ± 2.3 g) were randomly stocked into 12 tanks (25 fish/1,000-L tank). Control (AA_0_) and AA (AA_100_, AA_200_, and AA_400_) diets were randomly assigned to the three tanks. Fish were fed twice daily (09:00 and 18:00) for 12 weeks until apparent satiation. The feeding trials were conducted in a seawater (salinity: 32.3 ± 0.5 ppt) flow-through system with a flow rate of 6.5 L/min. Water temperature (17.5 ± 3.5°C), dissolved oxygen levels (8.3 ± 1.0 mg/l), salinity (32 ± 1 ppt), pH (7.4 ± 0.5), and photoperiod (14 h light: 10 h dark) were carefully maintained throughout the feeding trials.

### Sample Collection

At the end of the feeding trial, fish were starved for 24 h, and all surviving fish in the tanks were caught and weighed. Subsequently, three fish from each tank (nine fish/diet group) were anesthetized with tricaine methanesulfonate (MS-222; 100 mg/l, buffered to pH 7.4) for whole-body composition analysis. An equal number of fish was sampled for blood collection using heparinized syringes to quantify the plasma amylase activity.

### Variables Measured for Growth and Feed Utilization Parameters

At the start of the experiment, fish body weight was measured to calculate the initial body weight (IBW). During the feeding trials, the amount of supplemented feed in each tank was monitored to calculate the feed utilization parameters. At the end of the trial, the final body weight (FBW), weight gain (WG, %), specific growth rate (SGR), feed conversion ratio (FCR), protein efficiency ratio (PER), and survival rate (%) were calculated using the following equations:

IBW (g) = Initial weight of total fish in tank/Fish number

FBW (g)= Final weight of total fish in tank/Fish number

WG (%) = ([FBW – IBW]/IBW) × 100

SGR (%/day) = ([ln FBW – ln IBW]/day) × 100

FCR = Dry feed intake/Wet body weight gain

PER =Wet weight gain/Protein fed

Survival (%) = (number of fish at the end of the trial/number of fish at the beginning of the trial) × 100.

### Feed and Whole-Body Proximate Composition Analyses

Proximate composition analyses of the experimental feeds and whole fish bodies were conducted according to the standard methods of the Association of Official Analytical Chemists [26]. The collected fish samples were homogenized using an industrial food processor. Samples were dried in a convection oven at 105°C for 24 h to determine the moisture content. Crude protein content was determined using the Kjeldahl method (N × 6.25) after acid digestion with an auto Kjeldahl system (VAP50OT/TT125; Gerhardt GmbH & Co., Germany). Crude lipids were measured using the Soxhlet extraction method with the Tecator Soxtec System HT 1046 (Tecator AB, Sweden) after freeze-drying the samples for 20 h. Ash was analyzed by incineration at 550°C in a muffle furnace for 5 h. Feed energy content was determined using an isoperibol bomb calorimeter (Parr 6300; Parr Instrument Company Inc., USA).

### Apparent Digestibility and Fecal Particle Size Test

After sample collection, ADC of the dry matter, proteins, lipids, carbohydrates, and energy was determined for the remaining fish. Each experimental diet was fed to the olive flounder (18 fish/ 1,000-L tank) at apparent satiation twice daily for two weeks. Then, fecal samples were collected by siphoning onto a mesh two hours after feeding to avoid leaching of the nutrients and stored in tubes at −20°C until required for analysis. The fecal samples collected daily were pooled per tank (three experimental units/treatment) for ADC and fecal particle size analyses. ADC of the dry matter, protein, lipids, carbohydrates, and energy of the diets was calculated using the following equation given by Bureau *et al*. [27]:

ADC _diet_ = 1 − ([F/D] × [Di/Fi]),

where D = % nutrient of diet, F = % nutrient of feces, Di = % digestion indicator of diet, and Fi = % digestion indicator of feces.

Fecal particle size was measured using a Mastersizer 3000 (Malvern, USA) fitted with a Hydro LV wet sample measurement accessory that has detectors ranging from 0.01 to 10.000 μM. Feces were suspended in water in the Hydro LV and circulated through the Mastersizer 3000 for 25 consecutive measurements, each of which was 5 s in duration. The first 25 measurements represent the particle size distribution of the fecal casts. The particle size distributions with 10, 50, and 90% of the total volume were calculated using the Mastersizer software.

### Feed Degradation Rate

Feed degradation rate was determined using a modified version of the method described by Azarfar *et al*. [28]. To prepare the artificial stomach fluid, NaCl (5 g/l) and KCl (1.5 g/l) were added to distilled water containing 1 N HCl to adjust the pH to 3.0. Pellets for each experimental diet (6 g, each approximately 0.6 cm in diameter) were placed in a 2-L beaker containing 1 L of artificial stomach fluid at room temperature for 90 min without shaking. After soaking, each feed was poured onto a 200-μM sieve. The feed material retained by the sieve was collected and placed in a pre-weighed aluminum dish. The material was dried in a forced-air oven at 130°C for 2 h and weighed. Relative difference in the dry mass before and after 90 min of soaking against the original sample dry mass was calculated as the feed degradation rate. In another test to determine the feed degradation rate in the stomach, two fish from each group were sampled at the end of the digestibility trial. Their stomach pellets were collected in Petri dishes and photographed using a digital camera after 2, 4, and 8 h of feeding.

### Quantitative Reverse Transcription-Polymerase Chain Reaction (qRT-PCR)

To quantify the effects of α-amylase administration on the expression levels of immune- and growth-related genes in olive flounder, qRT-PCR was conducted as described by Hasan *et al*. [29] and Jang *et al*. [30], with some modifications. Briefly, 3 fish/tank (9 fish/group) were anesthetized to collect their intestinal and muscle samples. Total RNA was extracted from the samples using the GeneAll Hybrid-R RNA Isolation Kit (GeneAll Biotechnology, Korea), according to the manufacturer’s instructions. Residual genomic DNA was removed from the isolated RNA using the Riboclear Plus Kit (GeneAll Biotechnology). Next, RNA purity (260/280) and concentration (ng/μl) were assessed using the NanoDrop spectrophotometer (Thermo Scientific, USA), and 1 μg of purified RNA was used to prepare cDNA using the PrimeScript cDNA Synthesis Kit (Takara, Japan). All gene-specific primers (Table 2) were designed using the Primer3 software and 25 μl reaction mixtures were prepared with 2 μl cDNA (1:20 dilution), 9.5 μl distilled water, 12.5 μl SYBR Green, and 0.5 μl forward and reverse primers. PCR amplifications were performed using the following protocol: 30 s denaturation at 95°C, followed by 40 cycles of denaturation at 95°C for 5 s, and 30 s of annealing and extension at 60°C. In each sample, elongation factor-1α (Ct value of 21.95 ± 0.56) was used as a reference gene to standardize the results. Dissociation curves were generated at the end of PCR to assess the specificity of the reaction. Relative expression of growth-related genes was quantified using the 2^−ΔΔCT^ method with the Thermal Cycler Dice system (Model TP700/760; Takara, Japan) containing the V5.0x software.

### Plasma Amylase Activity

Collected blood was centrifuged at 5,000 rpm (rcf: 7,168 × *g*; VS-24SMTi; VISION Scientific, Co., Ltd., Korea) for approximately 10 min at 4°C to separate the plasma. The assay was performed immediately using an Amylase Alpha ELISA Kit (MyBiosource, USA), according to the manufacturer’s instructions. Briefly, 50 μl of plasma and 100 μl of horseradish peroxidase-conjugate reagent were added to every well. Covered plates were incubated for 60 min at 37°C and washed four times with the wash solution. A mixture of 100 μl chromogen solution and 50 μl stop solution was added to every well. The absorbance was measured over time at 450 nm using a microplate reader (Sunrise, Tecan, Austria). One unit (U) of amylase activity was defined as the amount of cleaved amylase.

### Statistical Analysis

Homogeneity of error variance was determined using Levene’s test, and the dependent variables were subjected to one-way analysis of variance using SAS Version 9.3 (SAS Institute, USA). When a significant (*p* < 0.05) treatment effect was observed, the Duncan post-hoc test was performed to compare the mean treatment values. Data are represented as the mean ± SD.

## Results

### Growth, Feed Utilization, and Whole-Body Proximate Composition

After 12 weeks of feeding trial, no significant differences (*p* ≥ 0.05) in FBW, WG%, and SGR (growth parameters) were observed among the groups. Moreover, a similar modulation pattern was observed in the feed utilization parameters (FCR and PER) and survival rate of the experimental olive flounder (Table 3). At the end of the feeding experiment, α-amylase treatment had no significant effects (*p* ≥ 0.05) on whole-body composition parameters, including the moisture, crude lipid, crude protein, and ash contents, of olive flounder (Table 4).

### ADC

Fish fed the experimental diets (AA_100_, AA_200_, and AA_400_) exhibited significant differences in carbohydrate ADC compared with the control (*p* < 0.05). However, the carbohydrate ADCs were not significantly different among the three treatment diets (*p* ≥ 0.05). Similarly, there were no significant differences in the dry matter, crude lipid, crude protein, and gross energy contents between the control and treated fish (Table 5).

### Feed Degradation Rate (%)

AA_0_ and AA_400_ groups exhibited the lowest and highest feed degradation rates (%), respectively. Compared to the control, α-amylase significantly and dose-dependently increased the feed degradation rates in AA_100_, AA_200_, and AA_400_ groups to 41.8 ± 2.5, 55.3 ± 1.2, and 81.7 ± 2.9%, respectively (Table 6).

**Fig. 1:**
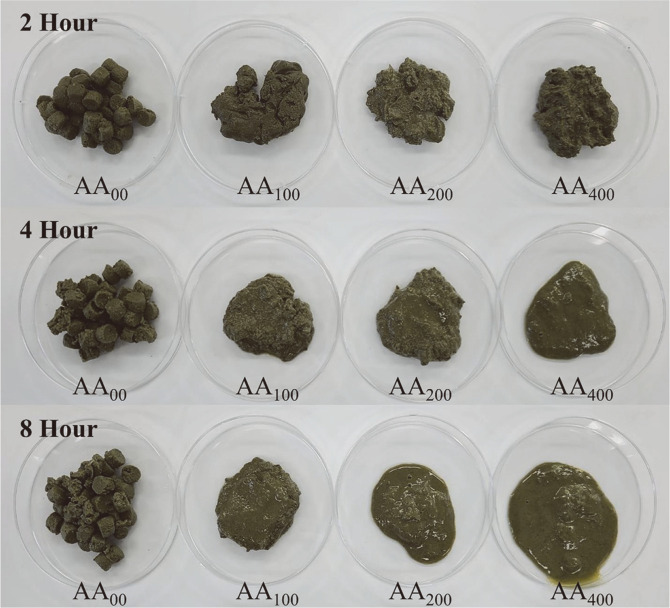
Photograph of the feed collected from flounder stomach after 2, 4, and 8 h of feeding supplemented with 0 (A_00_), 100 (AA_100_), 200 (AA_200_), and 400 (AA_400_) mg/kg of α-amylase.

Feed pellets collected from the fish stomachs were gradually degraded at 2, 4, and 8 h. Interestingly, this degradation was time-dependent and higher diet morphological alteration/degradation was observed in the group with the highest α-amylase concentration (Fig. 1).

### Plasma α-Amylase Concentration and Fecal Particle Size

**Fig. 2:**
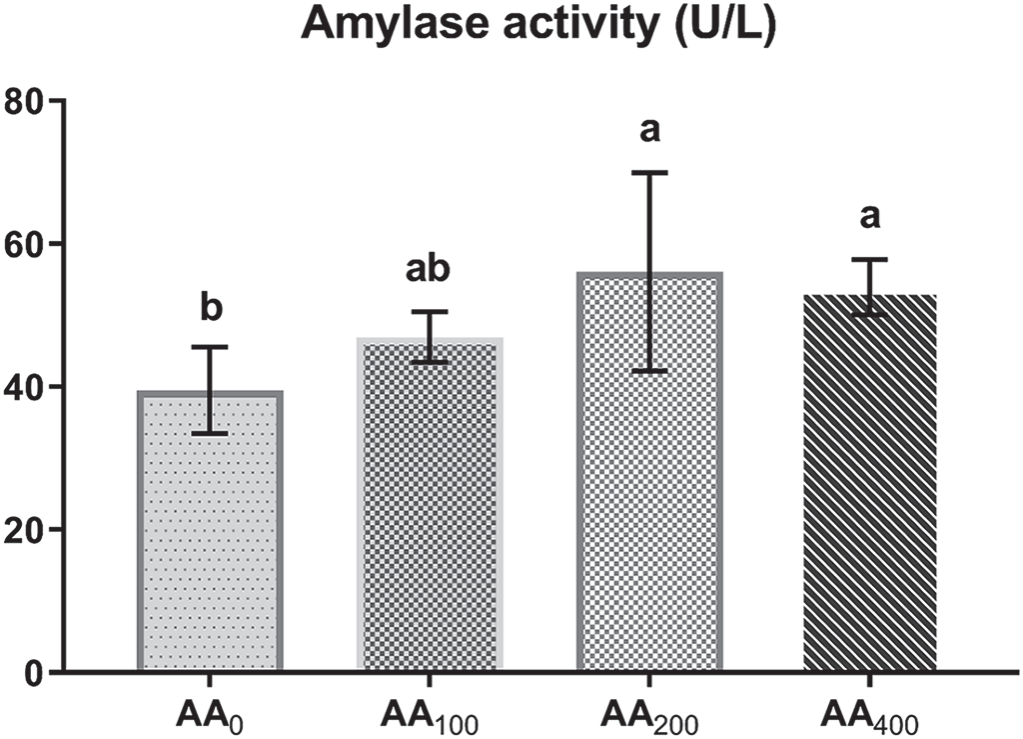
α-Amylase activity of plasma between the experimental groups. The data represent the mean ± standard deviation (6 fish/group); values with different letters indicate significant differences (*p* < 0.05).

After 12 weeks of feeding, plasma α-amylase concentration increased (*p* < 0.05) in both the AA_200_ and AA_400_ groups relative to that in the control. However, no significant differences were observed between AA_200_ and AA_400_ and between AA_0_ and AA_100_ groups (Fig. 2).

**Fig. 3:**
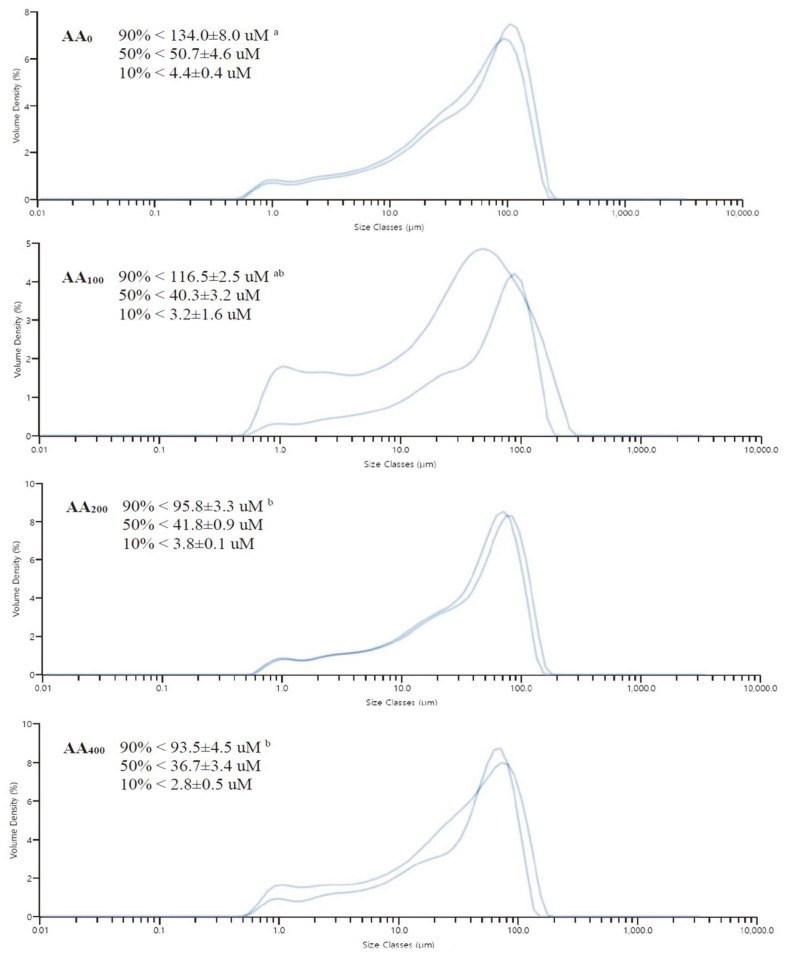
Fecal particle size of olive flounder fed with four types of experimental diets (*n* = 3 tanks per diet). Feed was supplemented with 0 (AA_00_), 100 (AA_100_), 200 (AA_200_), and 400 (AA_400_) mg/kg of α-amylase.

In the 90% fecal particle size class, the particle size of AA_0_ group was significantly larger than those of the AA_200_ and AA_400_ groups. Moreover, 90% fecal particle size in olive flounder fed with the control (AA_0_) diet was < 134.0 ± 8.0 μM, whereas the AA_100_, AA_200_, and AA_400_ groups exhibited particle sizes of < 116.5 ± 2.5, < 95.8 ± 3.3, and < 93.5 ± 4.5 μM, respectively (Fig. 3; Table 7). In contrast, no significant variations were observed in the 50 and 10% size classes. Additionally, 90% particle sizes of the AA_0_ and AA_100_ groups were similar, whereas those of AA_200_ and AA_400_ were significantly different (*p* < 0.05) from that of AA_0_.

### Effects of α-Amylase on the Transcription of Immune- and Growth-Related Genes

**Fig. 4:**
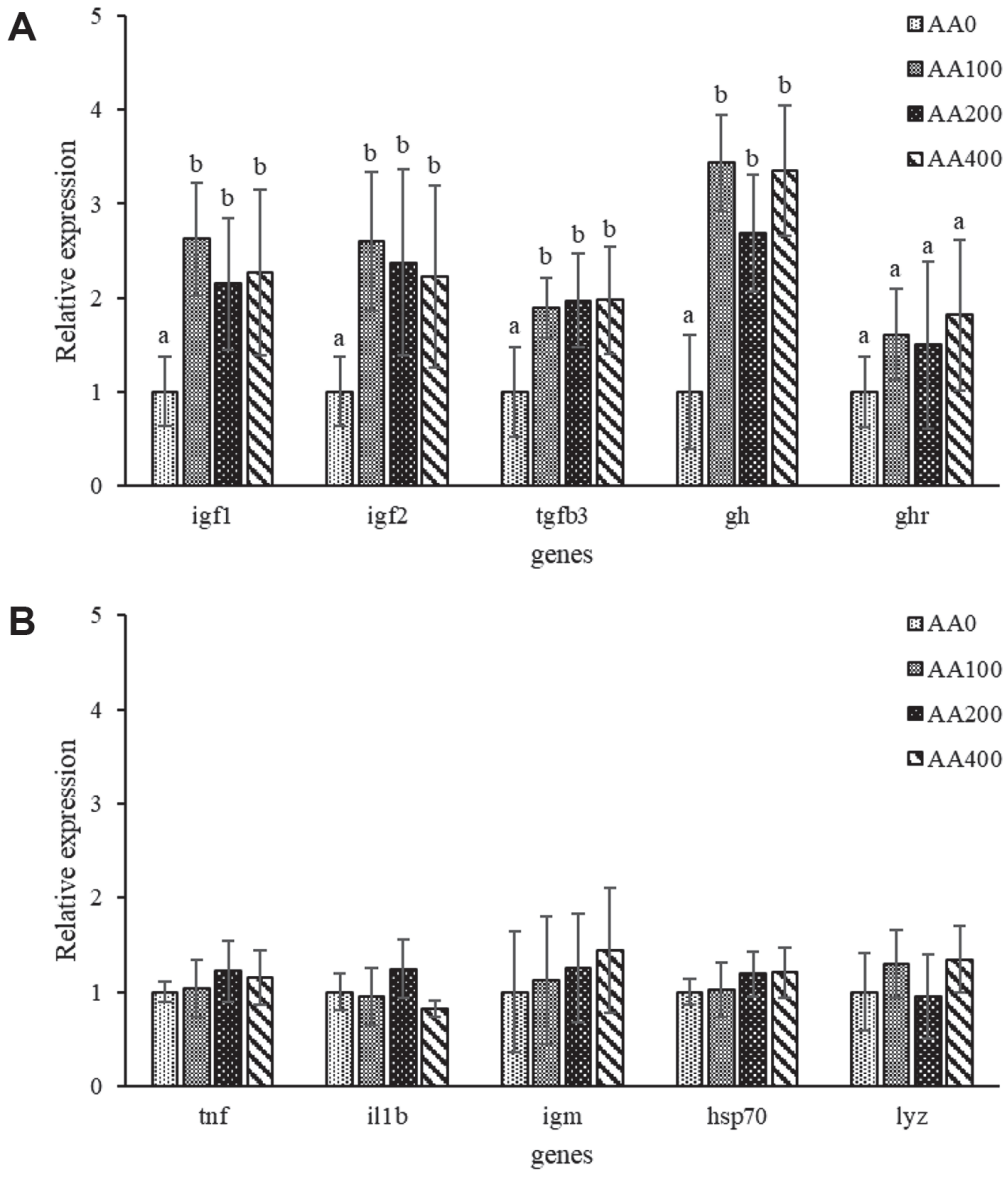
Profiles of gene expression in the muscle (A) and intestine (B) of olive flounder. Expression of these genes in olive flounder was measured by RT-qPCR after 12 weeks of feeding for the Control (AA_0_), AA_100_, AA_200_, and AA^400^ groups. Levels of gene expression were quantified relative to *elongation factor*-1α transcription. The data are represented as the means ± standard deviation (6 fish/group); means that do not share the same letter differ significantly (*p* < 0.05).

Compared with the control, transcription levels of seven immune-related genes (tumor necrosis factor [*TNF*]-*α*, interleukin [*IL*]-*1β*, *IL-2*, immunoglobulin [*Ig*]-*M*, heat shock protein [*HSP*]-*70*, lysozyme, and amylase alpha [*AMY*]-*2A*) remained unchanged in the intestine of the experimental olive flounders (*p* ≥ 0.05; Fig. 4A).

In contrast, expression levels of growth-related genes, such as insulin-like growth factor *(IGF)-1*, *IGF-2*, and transforming growth factor *(TGF)-β3* in olive flounder muscle were approximately 2–2.5 times higher (*p* < 0.05) in the α-amylase supplemented group than in the control (Fig. 4B). Moreover, growth hormone (*GH*) transcription was 3-fold higher in the AA_100_ and AA_400_ groups than in the control group, whereas no changes in the expression levels of GH receptor were observed among the experimental groups. Notably, expression levels of *IGF-1*, *IGF-2*, *TGF-β3*, and *GH* were identical among the AA_100_, AA_200_, and AA_400_ groups.

## Discussion

In this study, dietary supplementation with three concentrations of α-amylase had no significant effects on the growth, feed utilization, and whole-body composition of olive flounder. Previous studies reported that cellulase and an enzyme cocktail comprising xylanase, amylase, cellulase, protease, and β-glucanase have no effects on the growth performance of *Oreochromis niloticus* [24] and *O. mykiss* [25], respectively. A dietary multi-enzyme complex (glucanase, cellulose, xylanase, pentosanase, and phytase) increases the growth rate and feed efficiency in Japanese seabass (*Lateolabrax japonicas*) [31]. Addition of Kemzyme (a commercial enzyme cocktail) at 0.4, 1.2, and 3.6 g/kg to a cotton seed-containing diet (40% protein) did not significantly affect the growth performance of gilthead sea bream (*Sparus aurata*) [32]. Similarly, no improvements in growth performance were observed in *O. mykiss*, whose diet was supplemented with phytase to eliminate its phytic acid content [33]. Normally, dietary exogenous enzymes are supplemented to increase the feed utilization of fish and accelerate their growth. In contrast to our findings, other studies have reported that α-amylase supplementation improves the WG rates and feed utilization of *Oreochromis niloticus* [20], in addition to improving the flesh quality and feed utilization of Atlantic salmon (*Salmo salar*) [34]. Similar to our findings regarding the whole-body proximate composition of olive flounder, previous studies reported that the administration of phytase and a commercial enzyme mixture (protease, xylanase, and β-glucanase) has no significant effects on the moisture, protein, lipid, and ash contents of *S. salar* [35] and *Oreochromis niloticus* × *Oreochromis aureus* [36], respectively. Moreover, other studies have reported that exogenous enzyme supplementation does not significantly change the survival rate [24, 35] or the whole-body proximate composition of fish [37], which is consistent with our findings. In this study, exogenous α-amylase was not supplemented in a carbohydrate-rich diet, and the differences between our findings and those of previous reports may be due to species variations, lack of alternative ingredients in the feed, size of the olive flounder at the start of the experiment, food preferences and feeding habits of this fish, and differences in the type, number, and combination of supplemented enzymes.

Also in this study, α-amylase had no significant effect on the ADC of dry matter, crude protein, lipid, and gross energy contents. However, an improved carbohydrate ADC was observed in all treatment groups. ADC of Indian major carp catla (*Catla catla*) and rohu improved after amylase supplementation in a gelatinized corn-based diet [38]. Supplementation with Natustarch (α-amylase) [4] and (α-galactosidase) [8] increases the ADC of silver perch and rainbow trout fed with a dehulled lupin-containing diet, respectively. Dalsgaard *et al*. [13] supplemented three diets containing high levels of plant-based ingredients, such as soybean (344 g/kg), sunflower (246 g/kg), and rapeseed (264 g/kg), with β-glucanase (67 mg/kg), xylanase (208 mg/kg), and protease (228 mg/kg), respectively. The apparent nutrient digestibility of sunflower and rapeseed diets improved moderately but β-glucanase significantly improved the ADC of the soybean-containing diet. Similarly, our findings demonstrated that α-amylase improved the carbohydrate ADC, suggesting that this enzyme promotes carbohydrate digestion and utilization, thereby improving energy availability in olive flounder.

The carbohydrates (especially starch) that bind to the active site of the enzyme for digestion are hydrophilic [39], and our in vitro experiments suggested that the improvement in the carbohydrate degradation rates of olive flounder was due to the presence of α-amylase. This enzyme is also likely involved in changes in the feed structure in the stomach. However, further experiments are required to confirm these findings. Dietary supplementation with 200 and 400 mg/kg of α-amylase increased plasma amylase concentration in the experimental olive flounder. No previous study has estimated the activity of digestive enzymes in the blood in response to the application of exogenous enzymes. However, α-amylase application elevates glucose-6-phosphate dehydrogenase levels in the liver and blood of *L. rohita*, in addition to reducing the levels of glucose-6-phosphatase, fructose-1,6-bisphosphate, alanine, and aspartate aminotransferase [19]. Moreover, amylase administration in striped catfish (*Pangasianodon hypophthalmus*) significantly increased hematological parameters, such as red and white blood cells, lymphocytes, and hematocrit, suggesting that this enzyme possesses immunomodulatory effects [40]. Similarly, Hassaan *et al*. [41] reported that 3,750 U/kg xylanase increased the levels of digestive enzymes, such as chymotrypsin, amylase, and lipase, in the intestine. In addition to fish, the application of α-amylase-producing bacterial culture in water increases the serum and small intestinal amylase content of broiler chickens [42], which is consistent with our findings. In contrast, supplementation with exogenous amylase and protease decreases the secretion of these two enzymes in the pancreas of birds [43]. Flounder intestinal histology depicts well-organized goblet cells and microvilli as well as an intact epithelial barrier [44]. The presence of different digestive enzymes, such as amylase, lipase, trypsin [45], and chymotrypsin [46] was detected in the fish intestine. The gastrointestinal tract pH and the presence of enzyme inhibitors limit the potential benefits of exogenous enzyme application [47]. In the early stage, the fish intestine is neutral or slightly alkaline (6.7–7.1), but in the adult stage, the pH can be below 5 [48]; therefore, acid-resistant enzymes should be provided according to the life stage and diversity of the fish digestive system [16]. In fish, digestive enzyme administration effect analysis mainly focuses on growth and feed utilization, blood biochemistry, digestibility, and gut microbiota [6, 19], without considering the alteration of supplemented enzyme properties by the influence of the digestive system environment. Further studies are needed to elucidate the molecular mechanisms by which exogenous enzyme activities are modulated in the intestinal environment of carnivorous fish, including olive flounder. In addition, the effects of dietary enzymes on the activity and secretion of other important enzymes in fish intestines, blood, and other tissues should be investigated.

GH binds to GH receptors and stimulates the synthesis of IGF-1 in the liver [49]. Our findings demonstrated that the levels of *GH*, *TGF-β3*, and *IGF-1* were significantly upregulated in the muscle tissues of α-amylase-treated fish compared to those of the control. IGF is an essential hormone for the growth and development of carp [50] and the TGF-β family is composed of dendritic proteins that regulate the growth and differentiation of many cell types and skeletal muscle [51]. Although the expression of growth-related genes was upregulated, the growth parameters of the flounder were similar at the end of the experiment. Similar upregulation of growth-related gene expression in different organs has been reported in tilapia [52] and crucian carp (*Carassius auratus*) [53] after supplementation with proteases and cellulases, respectively. The fish intestine is an important organ that is involved in digestion and absorption. Enterocytes contain dendritic cells, and different types of receptors act as immunomodulatory response mediators. However, α-amylase supplementation had no effect on the expression of immune-related genes in the intestine of olive flounder, suggesting that this enzyme has no immunomodulatory properties.

In this study, olive flounder fed without α-amylase produced larger (90% size class) fecal particles than the 200 and 400 mg/kg enzyme-supplemented groups. Fecal particle size is a key indicator of digestion and mechanical stability of feces [54]. Starch is positively correlated with the production of larger fecal particles [55-57], whereas oligosaccharides in feed have little to no effect on the particle size or structure. Therefore, the larger fecal particles observed in the control group could be due to a higher level of starch in the digested food caused by a lack of amylase enzymes. In contrast, the two groups with the highest amylase doses produced smaller fecal particles, presumably because the carbohydrates in the feed were properly digested by the exogenous amylase. This also explains the increase in carbohydrate ADC observed in our experiment. Very few studies have assessed the effects of supplementation with exogenous enzymes (amylase, xylanase, cellulase, and gluconase) on fecal particle size in aquaculture species. A recent study by Welker *et al*. [58] demonstrated that soybean meal and soy protein concentrate produced undesirable particle sizes in rainbow trout, whereas the addition of guar gum alleviated this negative impact. Moreover, tilapia fecal matter quantity was significantly reduced when external enzymes were supplemented in FM-replaced feed [59].

## Conclusion

In this study, dietary application of α-amylase at 200 and 400 mg/kg improved the carbohydrate ADC, blood amylase content, feed degradation rate, and fecal particle size in olive flounder. Interestingly, α-amylase had no significant effects on the growth, feed utilization, and whole-body composition of this fish species. However, the effects of exogenous enzyme supplementation on the immunology, intestinal microbiome, serum biochemistry, and transcription of growth and digestive genes in cultured olive flounder require further elucidation in future studies. Moreover, the involvement of external enzymes in the activation of different physio-immunological pathways needs to be investigated in the future.

## Figures and Tables

**Table 1:** Ingredient composition of the experimental diets fed to olive flounder over a 12-week growth trial (% of DM basis).

Ingredients	Diet no. (AA supplementation level, U/kg diet)			
	AA_0_	AA_100_	AA_200_	AA_400_
Fishmeal	60.0			
Soybean meal	10.3			
Tapioca starch	10.0			
Wheat flour	11.5			
Fish oil	5.0			
Mineral mixture^1^	1.0			
Vitamin mixture^2^	1.0			
Choline	0.5			
Cr_2_O_3_	0.5			
α-amylase^3^	0	0.01	0.02	0.04
NaCl	0.2	0.19	0.18	0.16
Total	100	100	100	100
*Analyzed nutrients compositions (as-is basis)*				
Moisture (%)	4.63	5.02	4.73	4.86
Crude protein (%)	51.1	51.2	50.9	51.0
Crude lipid (%)	10.2	9.8	10.4	10.0
Crude ash (%)	13.8	13.8	13.9	13.6
Gross energy (cal/g)	4932	4925	4942	4926

^1^Mineral premix contained the following amount which were diluted in cellulose (g/kg premix): NaCl, 30.3; MgSO_4_-7H_2_O, 95.6; NaH_2_PO_4_-2H_2_O, 60.8; KH_2_PO_4_, 167.3; CaH_4_(PO_4_)_2_-H_2_O, 94.7; Ferric citrate, 20.7; ZnSO_4_-7H_2_O, 15.3; Ca-lactate, 212.8; CuCl, 0.14; AlCl_3_-6H_2_O, 0.105; KI, 0.105; Na_2_Se_2_O_3_, 0.01; MnSO_4_-H_2_O, 1.4; CoCl_2_-6H_2_O, 0.7.

^2^Vitamin premix contained the following amount which were diluted in cellulose (g/kg premix): L-ascorbic acid, 171.1; myoinositol, 181.8; DL-a-tocopheryl acetate, 18.9; niacin, 36.4; p-aminobenzoic acid, 18.2; Ca-D-pantothenate, 12.7; riboflavin, 9.1; thiamin hydrochloride, 2.7; pyridoxine hydrochloride, 1.8; menadione, 1.8; retinyl acetate, 0.73; folic acid, 0.68; D-biotin, 0.27; cholecalciferol, 0.003.

^3^α-amylase from *Aspergillus oryzae* (30 units/mg; Sigma Aldrich, St. Louis, USA

**Table 2:** Gene specific primers used to quantify relative gene expression.

Gene	Sense	Oligonucleotide sequence (5` to 3`)	Access No.
Elongation factor-1-α (Reference)	F	GAGGTCAAGTCTGTGGAGAT	AB915949
	R	GGTGGTTCAGGATGATGAC	
Tumor necrosis factor	F	CCCTATGAACTGTAACAGTTTG	AB040448
	R	GTCAGGTACTTAACCCTCAT	
Interleukin 1 β	F	TGCTACCAGACCTTCAACAT	AB070835
	R	TCTTTCCAGCAGACAGTGGT	
Interleukin 2	F	ACATACGTACTTCAAGCTATCG	KY307833
	R	GTAAAGATTCCACTTGGTCCA	
Immunoglobulin M	F	GCCTCCTTCTTCTGCTCTG	AB109029
	R	CCTCAGTGGATGTTGTGATT	
Heat shock protein 70	F	CAATGATTCTCAGAGGCAAG	DQ662230
	R	TATCTAAGCCGTAGGCAATC	
Insulin-like growth factor 1	F	ATGTCTAGCGCTCTTTCCTT	AF061278
	R	CTTCTTGTTTTTTGTCTTGTCTG	
Insulin-like growth factor 2	F	AGAACCGTGGGATCGTAGA	AF091454
	R	TGCCACACCTCGTATTTG	
Transforming growth factor β 3	F	TCCAAGGTATTCCGCTTCAA	XM_020085122
	R	TTTGGCTTTGGGGTCATCT	
Growth hormone	F	TCCTCTCAGCCAATCACAGA	M23439
	R	TACGTCTCCACCTTGTGCAT	
Growth hormone receptor	F	CCACAAACTGGAAATCATTGG	AB058418
	R	CGAAAACAAGAACAACTGTGAG	

**Table 3:** Growth and feed utilization parameters of olive flounder fed with experimental diets for 12 weeks^1^.

	AA_0_	AA_100_	AA_200_	AA_400_	*P* value
IBW^2^	273.2±3.9	273.5±3.4	272.2±1.8	273.3±0.6	0.939
FBW^3^	555.3±4.5	564.5±8.4	563.8±2.3	566.1±7.1	0.203
FI^4^	331.4±4.3	323.7±7.1	329.3±3.3	326.1±4.6	0.312
WG (%)^5^	103.3±2.1	106.4±1.4	107.1±2.2	107.1±2.3	0.126
SGR^6^	1.01±0.01	1.04±0.01	1.04±0.02	1.04±0.02	0.123
FCR^7^	1.16±0.02	1.11±0.04	1.10±0.02	1.11±0.02	0.054
PER^8^	1.66±0.06	1.76±0.06	1.73±0.04	1.76±0.03	0.117
Survival (%)	98.7±2.3	100	97.3±2.3	100	0.219

^1^Values are mean ± SD of three replicates (3 tank/group). Values without superscript letters within the same row in the table are not significantly (*p* ≥ 0.05) different.

^2^IBW: Initial body weight (g); ^3^FBW: Final body weight (g); ^4^FI: Feed intake (g/fish); ^5^WG: Weight gain (%); ^6^SGR: Specific growth rate (%/d); ^7^FCR: Feed conversion ratio; ^8^PER: Protein efficiency ratio

**Table 4:** Whole-body proximate composition (%, as-is basis) of juvenile rainbow trout fed experimental diets for 12 weeks^1^.

	AA_0_	AA_100_	AA_200_	AA_400_	*P* value
Moisture	71.2±0.3	71.1±0.2	71.3±0.2	71.2±0.2	0.761
Crude protein	19.2±0.2	19.2±0.1	19.4±0.2	19.2±0.2	0.518
Crude lipid	5.02±0.08	5.12±0.09	4.92±0.11	5.07±0.13	0.326
Crude ash	3.43±0.18	3.36±0.06	3.52±0.28	3.51±0.19	0.834

^1^Values are mean ± SD of three replicates (3 tank/group). Values without superscript letters within the same row in the table are not significantly (*p* ≥ 0.05) different.

**Table 5:** Apparent digestibility coefficients (%) of dry matter, crude protein, crude lipid, carbohydrate, and energy for experimental diets fed to olive flounder for 12 weeks^1^.

	AA_0_	AA_100_	AA_200_	AA_400_	*P* value
Dry matter	69.3±0.9	72.6±0.5	71.8±0.3	72.6±1.1	0.103
Crude protein	87.4±0.4	87.1±0.2	86.8±0.2	87.5±0.5	0.600
Crude lipid	89.5±0.3	89.6±0.2	90.0±0.1	90.4±0.4	0.217
Carbohydrate	83.2±0.5^b^	88.7±0.2^a^	87.7±0.1^a^	88.6±0.5^a^	0.001
Gross energy	86.6±0.4	86.3±0.3	86.0±0.2	86.2±0.6	0.680

^1^Values are mean ± SD of three replicated tanks. Values without/similar and different superscript letters within the same row in the table are not significantly (*p* ≥ 0.05) and are significantly (*p* < 0.05) different, respectively.

**Table 6:** Feed degradation rate (%) in different experimental feeds^1^.

	AA_0_	AA_100_	AA_200_	AA_400_	*p*-value
Prepared pellet (g)	6.00	6.00	6.00	6.00	1.000
Soaked pellet (g)	4.71±0.14^a^	3.49±0.15^b^	2.68±0.07^c^	1.10±0.17^d^	<0.001
Feed degradation rate (%)^2^	21.5±2.3^d^	41.8±2.5^c^	55.3±1.2^b^	81.7±2.9^a^	<0.001

^1^Values are mean ± SD (3 times/group). Values without and different superscript letters within the same row in the table are not significantly (*p* ≥ 0.05) and are significantly (*p* < 0.05) different, respectively.

^2^Feed degradation rate (%): (dry pellet before soaking - dry pellet after soaking) / dry pellet before soaking × 100.

**Table 7:** Fecal particle size of olive flounder fed with four types of experimental diets for 12 weeks^1^.

Diet group	Particle size (µM)		
	10% size class	50% size class	90% size class
AA_0_	< 4.4±0.4^a^	< 50.7±4.6^a^	< 134.0±8.8^a^
AA_100_	< 3.2±1.6^a^	< 40.3±3.2^a^	< 116.5±2.5^ab^
AA_200_	< 3.8±0.10^a^	< 41.8±0.9^a^	< 95.8±3.3^b^
AA_400_	< 2.8±0.5^a^	< 36.7±3.4^a^	< 93.5±4.5^b^

^1^Values are mean ± SD (3 tanks/group). Values with same and different superscript letters within the same column in the table are not significantly (*p* ≥ 0.05) and are significantly (*p* < 0.05) different, respectively.

## References

[1] FAO Fisheries & Aquaculture-Species Fact Sheets-*Paralichthys olivaceus* (Temminck & Schlegel, 1846) (2021). Food and Agriculture Organization of the United Nations.

[2] FishStatJ-FAO Fisheries and Aquaculture Software (2020). [computer software]. FAO fisheries division. (Updated September 14, 2020).

[3] Suresh S, Suriyavathana M (2011). Carbohydrate characterization of the cassava varieties (co5 and h226). J. Dairy. Food Home Sci..

[4] Stone DAJ, Allan GL, Anderson AA (2003). Carbohydrate utilization by juvenile silver perch, *Bidyanus bidyanus* (Mitchell). III. The protein‐sparing effect of wheat starch‐based carbohydrates. Aquac. Res..

[5] Wilson RP (1994). Utilization of dietary carbohydrate by fish. Aquaculture.

[6] Kumar S, Chakravarty S (2018). Amylases. Enzymes in human and animal nutrition (Ed. C.S. Nunes and V. Kumar).

[7] Zhao W, Wei HL, Wang ZQ, He XS, Niu J (2022). Effects of dietary carbohydrate levels on growth performance, body composition, antioxidant capacity, immunity, and liver morphology in *Oncorhynchus mykiss* under cage culture with flowing freshwater. Aqua. Nut..

[8] Farhangi M, Carter CG (2007). Effect of enzyme supplementation to dehulled lupin‐based diets on growth, feed efficiency, nutrient digestibility and carcass composition of rainbow trout, *Oncorhynchus mykiss* (Walbaum). Aquac. Res..

[9] Takeuchi T, Jeong KS, Watanabe T (1990). Availability of extruded carbohydrate ingredients to rainbow trout *Oncorhynchus mykiss* and carp *Cyprinus carpio*. Bul. Japanese Soc..

[10] Qu H, Ke W, Wen Z, Guo B, Lu X, Zhao Y, Yang Y (2022). Effects of dietary carbohydrate on growth, feed utilization, hepatic glucose and lipid metabolism in endangered Yangtze sturgeon (Acipenser dabryanus). Aquac. Rep..

[11] Vásquez‐Torres W, Arias‐Castellanos JA (2013). Effect of dietary carbohydrates and lipids on growth in cachama (*Piaractus brachypomus*). Aquac. Res..

[12] Cowieson AJ, Hruby M, Pierson EEM (2006). Evolving enzyme technology: impact on commercial poultry nutrition. Nutr. Res. Rev..

[13] Dalsgaard J, Verlhac V, Hjermitslev NH, Ekmann KS, Fischer M, Klausen M (2012). Effects of exogenous enzymes on apparent nutrient digestibility in rainbow trout (*Oncorhynchus mykiss*) fed diets with high inclusion of plant-based protein. Anim. Feed Sci. Technol..

[14] Jiang TT, Feng L, Liu Y, Jiang WD, Jiang J, Li SH (2014). Effects of exogenous xylanase supplementation in plant protein‐enriched diets on growth performance, intestinal enzyme activities and microflora of juvenile Jian carp (*Cyprinus carpio* var. Jian). Aquac. Nutr..

[15] Adeola O, Cowieson AJ (2011). Board-invited review: opportunities and challenges in using exogenous enzymes to improve nonruminant animal production. J. Anim. Sci..

[16] Liang Q, Yuan M, Xu L, Lio E, Zhang F, Mou H (2022). Application of enzymes as a feed additive in aquaculture. Mar. Life. Sci. Technol..

[17] LP Information Inc, 2022 Global animal feed enzymes market growth 2022-2028 Market Research.com.

[18] Upreti A, Byanju B, Fuyal M, Chhetri A, Pandey P, Ranjitkar R (2019). Evaluation of α-amylase, lipase inhibition and in-vivo pharmacological activities of *Eucalyptus camaladulensis* Dehnh leaf extract. J. Trad Complement. Med..

[19] Kumar S, Sahu NP, Pal AK, Sagar V, Sinha AK, Baruah K (2009). Modulation of key metabolic enzyme of *Labeo rohita* (Hamilton) juvenile: effect of dietary starch type, protein level and exogenous α-amylase in the diet. Fish Physiol. Biochem..

[20] Goda AMA, Mabrouk HAHH, Wafa MAEH, El-Afifi TM (2012). Effect of using baker's yeast and exogenous digestive enzymes as growth promoters on growth, feed utilization and hematological indices of Nile tilapia, *Oreochromis niloticus* fingerlings. J. Agri. Sci. Tech. B.

[21] Zamini A, Kanani HG, azam Esmaeili A, Ramezani S, Zoriezahra SJ (2014). Effects of two dietary exogenous multi-enzyme supplementation, Natuzyme^®^ and betamannanase (Hemicell^®^), on growth and blood parameters of Caspian salmon (*Salmo trutta caspius*). Comp. Clin. Pathol..

[22] Yildirim YB, Turan F (2010). Effects of exogenous enzyme supplementation in diets on growth and feed utilization in African catfish, Clarias gariepinus. J. Anim. Vet. Adv..

[23] Zhou Y, Yuan X, Liang XF, Fang L, Li J, Guo X (2013). Enhancement of growth and intestinal flora in grass carp: the effect of exogenous cellulase. Aquaculture.

[24] Yigit NO, Olmez M (2011). Effects of cellulase addition to canola meal in tilapia (*Oreochromis niloticus* L.) diets. Aquac. Nutr..

[25] Ogunkoya AE, Page GI, Adewolu MA, Bureau DP (2006). Dietary incorporation of soybean meal and exogenous enzyme cocktail can affect physical characteristics of faecal material egested by rainbow trout (*Oncorhynchus mykiss*). Aquaculture.

[26] AOAC (Association of Official Analytical Chemists) (1995). Official Methods of Analysis, sixteenth ed..

[27] Bureau DP, Harris AM, Cho CY (1999). Apparent digestibility of rendered animal protein ingredients for rainbow trout (*Oncorhynchus mykiss*). Aquaculture.

[28] Azarfar A, Tamminga S, Boer H (2007). Effects of washing procedure, particle size and dilution on the distribution between non‐washable, insoluble washable and soluble washable fractions in concentrate ingredients. J. Sci. Food Agric..

[29] Hasan MT, Jang WJ, Lee BJ, Kim KW, Hur SW, Lim SG (2019). Heat killed *Bacillus* sp. SJ-10 probiotic acts as a growth and humoral innate immunity response enhancer in olive flounder (*Paralichthys olivaceus*). Fish Shellfish Immunol..

[30] Jang WJ, Lee SJ, Jeon MH, Kim TY, Lee JM, Hasan MT (2021). Characterization of a *Bacillus* sp. KRF-7 isolated from the intestine of rockfish (*Sebastes schlegelii*) and effects of dietary supplementation in the aquaculture industry. Fish Shellfish Immunol..

[31] Ai Q, Mai K, Zhang W, Xu W, Tan B, Zhang C (2007). Effects of exogenous enzymes (phytase, non-starch polysaccharide enzyme) in diets on growth, feed utilization, nitrogen and phosphorus excretion of Japanese seabass, *Lateolabrax japonicus*. Comp. Biochem. Physiol. A.

[32] Deguara S, Jauncey K, Feord J, Lopez J (1999). Growth and feed utilization of gilthead sea bream, Sparus aurata, fed diets with supplementary enzymes. Feed Manufacturing in the Mediterranean Region: Recent Advances in Research and Technology (Ed. J. Brufau and A. Tacon).

[33] Vielma J, Mäkinen T, Ekholm P, Koskela J (2000). Influence of dietary soy and phytase levels on performance and body composition of large rainbow trout (*Oncorhynchus mykiss*) and algal availability of phosphorus load. Aquaculture.

[34] Carter CG, Houlihan DF, Buchanan B, Mitchell AI (1994). Growth and feed utilization efficiencies of seawater Atlantic salmon, Salmo salar L, fed a diet containing supplementary enzymes. Aquac. Res..

[35] Sajjadi M, Carter CG (2004). Dietary phytase supplementation and the utilisation of phosphorus by Atlantic salmon (*Salmo salar* L.) fed a canola-meal-based diet. Aquaculture.

[36] Lin S, Mai K, Tan B (2007). Effects of exogenous enzyme supplementation in diets on growth and feed utilization in tilapia, *Oreochromis niloticus* x *O. aureus*. Aquac. Res..

[37] Ng WK, Chong KK (2002). The nutritive value of palm kernel and the effect of enzyme supplementation in practical diets for red hybrid tilapia (*Oreochromis* sp). Asian Fish. Sci..

[38] Kumar S, Sahu NP, Pal AK, Choudhury D, Mukherjee SC (2006). Studies on digestibility and digestive enzyme activities in *Labeo rohita* (Hamilton) juveniles: effect of microbial α-amylase supplementation in non-gelatinized or gelatinized corn-based diet at two protein levels. Fish Physiol. Biochem..

[39] Liu QZ, Zhang H, Dai HQ, Zhao P, Mao YF, Chen KX (2021). Inhibition of starch digestion: the role of hydrophobic domain of both α-amylase and substrates. Food Chem..

[40] Khalil M, Azmat H, Khan N, Javid A, Hussain A, Hussain S.M (2018). Growth responses of striped catfish *Pangasianodon hypophthalmus* (Sauvage, 1878) to exogenous enzyme added feed. Pakistan J. Zool..

[41] Hassaan MS, Mohammady EY, Soaudy MR, Abdel Rahman AAS (2019). Exogenous xylanase improves growth, protein digestibility and digestive enzymes activities in Nile tilapia, *Oreochromis niloticus*, fed different ratios of fish meal to sunflower meal. Aquac. Nutr..

[42] Onderci M, Sahin N, Sahin K, Cikim G, Aydin A, Ozercan I (2006). Efficacy of supplementation of α-amylase-producing bacterial culture on the performance, nutrient use, and gut morphology of broiler chickens fed a corn-based diet. Poult. Sci..

[43] Mahagna M, Nir I, Larbier M, Nitsan Z (1995). Effect of age and exogenous amylase and protease on development of the digestive tract, pancreatic enzyme activities and digestibility of nutrients in young meat-type chicks. Reprod. Nutr. Dev..

[44] Hasan MT, Jang WJ, Lee BJ, Hur SW, Lim SG, Kim KW (2021). Dietary supplementation of *Bacillus* sp. SJ-10 and *Lactobacillus plantarum* KCCM 11322 combinations enhance growth and cellular and humoral immunity in olive flounder (*Paralichthys olivaceus*). Probiotics Antimicrob. Proteins.

[45] Jang WJ, Lee JM, Hasan MT, Lee BJ, Lim SG, Kong IS (2019). Effects of probiotic supplementation of a plant-based protein diet on intestinal microbial diversity, digestive enzyme activity, intestinal structure, and immunity in olive flounder (*Paralichthys olivaceus*). Fish Shellfish Immunol..

[46] Seo BS, Park SJ, Hwang SY, Lee YI, Lee SH, Hur SW (2022). Effects of decreasing fishmeal as main source of protein on growth, digestive physiology, and gut microbiota of olive flounder (*Paralichthys olivaceus*). Animals.

[47] Castillo S, Gatlin III DM (2015). Dietary supplementation of exogenous carbohydrase enzymes in fish nutrition: a review. Aquaculture.

[48] Mahr K, Grabner M, Hofer R, Moser H (1983). Histological and physiological development of the stomach in *Coregonus* sp. Arch. Hyd-robiol..

[49] Reindl KM, Kittilson JD, Bergan HE, Sheridan MA (2011). Growth hormone-stimulated insulin-like growth factor-1 expression in rainbow trout (*Oncorhynchus mykiss*) hepatocytes is mediated by ERK, PI3K-AKT, and JAK-STAT. Am. J. Physiol. Regul. Integr. Comp. Physiol..

[50] Cao YB, Chen XQ, Wang S, Chen XC, Wang YX, Chang JP (2009). Growth hormone and insulin-like growth factor of naked carp (*Gymnocypris przewalskii*) in Lake Qinghai: expression in different water environments. Gen. Comp. Endocrinol..

[51] Funkenstein B, Olekh E, Jakowlew SB (2010). Identification of a novel transforming growth factor-β (TGF-β6) gene in fish: regulation in skeletal muscle by nutritional state. BMC Mol. Biol..

[52] Hassaan MS, El-Sayed AIM, Soltan MA, Iraqi MM, Goda AM, Davies SJ (2019). Partial dietary fish meal replacement with cotton seed meal and supplementation with exogenous protease alters growth, feed performance, hematological indices and associated gene expression markers (GH, IGF-I) for Nile tilapia, *Oreochromis niloticus*. Aquaculture.

[53] Shi X, Luo Z, Chen F, Wei CC, Wu K, Zhu XM (2017). Effect of fish meal replacement by Chlorella meal with dietary cellulase addition on growth performance, digestive enzymatic activities, histology and myogenic genes' expression for crucian carp *Carassius auratus*. Aquac. Res..

[54] Brinker A (2007). Guar gum in rainbow trout (*Oncorhynchus mykiss*) feed: The influence of quality and dose on stabilisation of faecal solids. Aquaculture.

[55] Khorrami B, Kheirandish P, Zebeli Q, Castillo-Lopez E (2022). Variations in fecal pH and fecal particle size due to changes in dietary starch: Their potential as an on-farm tool for assessing the risk of ruminal acidosis in dairy cattle. Res. Vet. Sci..

[56] Amirkolaie AK, Verreth JA, Schrama JW (2006). Effect of gelatinization degree and inclusion level of dietary starch on the characteristics of digesta and faeces in Nile tilapia (*Oreochromis niloticus* (L.)). Aquaculture.

[57] do Carmo Gominho-Rosa M, Rodrigues APO, Mattioni B, de Francisco A, Moraes G, Fracalossi DM (2015). Comparison between the omnivorous jundiá catfish (*Rhamdia quelen*) and Nile tilapia (*Oreochromis niloticus*) on the utilization of dietary starch sources: Digestibility, enzyme activity and starch microstructure. Aquaculture.

[58] Welker TL, Liu K, Overturf K, Abernathy J, Barrows FT (2021). Effect of soy protein products and gum inclusion in feed on fecal particle size profile of rainbow trout. Aquac. J..

[59] Soltan MA (2009). Effect of dietary fish meal replacement by poultry by-product meal with different grain source and enzyme supplementation on performance, feces recovery, body composition and nutrient balance of Nile tilapia. Pak. J. Nutr..

